# ASSOCIATION OF NUTRITIONAL STATUS WITH LUNG FUNCTION AND MORBIDITY IN CHILDREN AND ADOLESCENTS WITH CYSTIC FIBROSIS: A 36-MONTH COHORT STUDY

**DOI:** 10.1590/1984-0462/;2018;36;1;00006

**Published:** 2018-01-15

**Authors:** Daniela Barbieri Hauschild, Anauã Franco Rosa, Julia Carvalho Ventura, Eliana Barbosa, Emília Addison Machado Moreira, Norberto Ludwig, Yara Maria Franco Moreno

**Affiliations:** a Universidade Federal de Santa Catarina, Florianópolis, SC, Brasil.; b Hospital Infantil Joana de Gusmão, Florianópolis, SC, Brasil.

**Keywords:** Cystic fibrosis, Nutritional status, Spirometry, Cohort studies, Pediatrics, Fibrose cística, Estado nutricional, Espirometria, Estudos de coortes, Pediatria

## Abstract

**Objective:**

To evaluate the association between nutritional status, lung function and morbidity in a 36-month cohort in children and adolescents with cystic fibrosis.

**Methods:**

Prospective cohort of children and adolescents with cystic fibrosis aged 1-15 years. At the baseline, the nutritional status was determined by weight-for-height and body mass index-for-age for children <2 years and ≥2 years, respectively, and classified as: nutritional failure, nutritional risk and acceptable; and by the 50th percentile, according to the World Health Organization (WHO) growth charts. Lung function was assessed by forced expiratory volume in one second (FEV1). Morbidity was determined by the presence of infection and hospitalization by pulmonary exacerbation. Risk ratio and 95% confidence interval (95%CI) were calculated, being significant when p<0.05.

**Results:**

We evaluated 38 children and adolescents (median age 3.8 years). Patients that were classified as having nutritional failure at baseline had a RR of 5.00 (95%CI 1.49; 16.76) to present impaired lung function after 36 months. Those classified bellow the 50th percentile had a RR of 4.61 (95%CI 0.89; 23.81) to present the same outcome. Nutritional status was not a risk factor for morbidity in this cohort.

**Conclusions:**

Nutritional deficit was associated with impaired lung function, but not with morbidity in children and adolescents with cystic fibrosis.

## RESUMO

**Objetivo:**

Avaliar a associação entre estado nutricional, função pulmonar e morbidade em coorte de 36 meses de crianças e adolescentes com fibrose cística (FC).

**Métodos:**

Coorte prospectiva de 36 meses, com crianças e adolescentes com FC e idade entre 1 e 15 anos. No tempo inicial, o estado nutricional foi determinado a partir dos indicadores: peso-para-estatura e índice de massa corporal-para-idade, para crianças <2 anos e ≥2 anos, respectivamente, e classificado em: falência nutricional, risco nutricional e estado nutricional aceitável; também foi determinado por meio do percentil 50°, de acordo com a curva de crescimento da *World Health Organization* (WHO). A função pulmonar foi avaliada pelo volume expiratório forçado no primeiro segundo (VEF_1_). A morbidade foi determinada pela presença de infecção e hospitalização por exacerbação pulmonar. Foi calculado risco relativo (RR) e intervalo de confiança (IC95%), considerando significante p<0,05.

**Resultados:**

Foram avaliadas 38 crianças e adolescentes (mediana de idade 3,8 anos). Os pacientes classificados em falência nutricional no início do estudo mostraram um RR de 5,00 (IC95% 1,49; 16,76) para o comprometimento da função pulmonar após 36 meses. Aqueles classificados abaixo do percentil 50° apresentaram RR de 4,61 (IC95% 0,89; 23,81) para o desfecho. O estado nutricional não foi fator de risco para morbidade.

**Conclusões:**

O déficit nutricional esteve associado ao comprometimento da função pulmonar, mas não com a morbidade em crianças e adolescentes com FC.

## INTRODUCTION

Cystic fibrosis (CF) is an autosomal recessive disorder, whose defect is found in the chromosome that codify the protein Cystic Fibrosis Transmembrane Conductance Regulator (CFTR). It is characterized by systemic manifestations, infections, obstruction of the respiratory system, pancreatic insufficiency and its nutritional repercussions.^1^

Lung disease is the main cause of morbidity and mortality in CF. In the airways, the reduction in the absorption of chloride and the increasing absorption of sodium result in mucosal changes, making it thicker and more viscous, which damages mucociliary clearance and leads to an environment that is favorable to bacterial colonization, especially by *Staphylococcus aureus* (*S. aureus*), *Pseudomonas aeruginosa* (*P*. *aeruginosa)*, *Haemophilus influenzae (H. influenzae)* and *Burkholderia cepacia (B. cepacia)*. The exacerbations of lung disease are common and manifest clinically through cough, dyspnea, anorexia and weight loss, and the reduction of spirometric parameters.^2^

The infections resulting from respiratory airways, the chronic inflammatory process and the poor absorption caused by pancreatic insufficiency trigger a picture of nutritional depletion, which may intervene in the prognosis of lung disease.^3^ Malnutrition in CF is associated with the deterioration of the lung function, being considered as a determining agent in the evolution of CF.^5^

The anthropometric parameters mostly used to assess the nutritional status of children and adolescents in CF, because they are associated with lung function and survival, are the weight-for-height (W/H) and body mass index-for-age (BMI/A) indicators. Currently, it is recommended that children and adolescents with CF can maintain the BMI/A ≥percentile 50°.^6^

Therefore, considering that the nutritional status can influence lung function and the clinical evolution of CF, this study aimed to assess the association between nutritional status with lung function and morbidity in a cohort of children and adolescents with CF, followed-up for 36 months in a reference center in the South of Brazil.

## METHOD

A prospective 36-month cohort study, conducted between 2009 and 2012, composed of children and adolescents being followed-up in the Interdisciplinary Outpatient Clinic of Cystic Fibrosis in a reference center for the treatment of CF in the state of Santa Catarina, Brazil.

It included children and adolescents aged between 1 and 15 years old, diagnosed with CF through the sweat test (chloride >6 mmol/L).^7^ The exclusion criteria were:

- Presence of pulmonary exacerbation;
- Use of antibiotics;
- Fever;
- Trauma or psychiatric disorder at the time of the first collection

In the beginning of the study, the anthropometric data were assessed by the researcher, and afterwards, every six months, by the medical records, as well as the data regarding lung function and morbidity. The outpatient clinic has a previously established protocol that guarantees the quality of the registered information. The patients using pancreatic enzymes were considered with pancreatic insufficiency. The Shwachman-Kulczycki^8^ score was used to classify the clinical condition and the severity of the disorder.

The project was approved by the Human Research Ethics Committee (# 048/2009). All legal parties in charge of the patient signed the informed consent form after the acceptance of patients and tutors. 

The anthropometric measurements were collected by nutritionists, physicians or Nursing technicians, members of the multiprofessional team in the outpatient clinic, according to the protocol proposed by the World Health Organization (WHO, 1995).^9^ The nutritional status was determined based on the W/H indicators in children <2 years of age, or BMI/A in children aged ≥2 years, and height-for-age (H/A) for all children. Body mass index (BMI) was obtained by dividing the weight by the height squared (kg/m^2^).

The weight measurement in children aged <2 years was carried out in a pediatric digital scale, Filizola^®^ (Santo André, São Paulo, Brazil), with a 0.01 kg interval and maximum capacity of 15.0 kg. For children aged ≥2 years, the weight was measured in a Balmak^®^ scale, model BK 50 F (Santa Bárbara d’Oeste, São Paulo, Brazil), with 0.1 kg accuracy and maximum capacity of 150.0 kg. The length of the children aged <2 years was assessed with a Sanny^®^ mobile stadiometer for children (São Paulo, São Paulo, Brazil), graded from 0 to 15 cm and with 1 mm of accuracy. The height of the children aged ≥2 years was observed using the anthropometer Alturaexata^®^ (Belo Horizonte, Minas Gerais, Brasil), with a 0.1 cm precision-scale.

The percentiles of the anthropometric indicators H/A, W/H and BMI/A were calculated using the reference curves proposed by the WHO (2006, 2007).^10^ ^,^ ^11^Based on the indicators of W/H, for children aged <2 years, or BMI/A for children aged ≥2 years, the children and adolescents were classified in: nutritional failure (<percentile 10°), nutritional risk (percentile 10-25°) or acceptable nutritional status (>percentile 25°). The children and adolescents with CF were also classified according to the cutoff point of percentile 50°.^12^ Low height was considered when the indicator H/A was <percentile 5°.^13^

The evaluation of lung function, conducted by a trained professional, was carried out based on the spirometric test (spirometer Puritan-Bennett Corporation^®^, model *Renaissance Spirometry System*, Wilnington-NC, USA) in children aged ≥6 years. Lung function was considered to be impaired when values of forced expiratory volume in the first second (FEV_1_)<70%, once these patients seem to be at higher risk of FEV_1_ reduction.^14^

The analysis of morbidity was conducted according to the number of hospitalizations per pulmonary exacerbation in the 36-month period and the presence of infection. The presence of infection by *P. aeruginosa, S. aureus* and *B. cepacia* was assessed by the analysis of oropharyngeal secretion. The samples of this secretion were obtained in the morning - using a sterile swab introduced in the oropharyngeal cavity - and processed after collection.^15^ The evaluation was made with microscopy (NIKON E200 microscope, Chiyoda/Toq, Japan), using the Gram method. The counting ≥ 10^4^ UFC/mL characterized the presence of infection.^15^ ^,^ ^16^

The data were analyzed in the statistical software STATA^®^ version 11.0 (College Station, Texas, USA), and the GraphPad Prism *trial version* (GraphPad Software, Inc., La Jolla, CA, USA). The continuous variables were expressed as mean and standard deviation, or median and interquartile range (IQR). The categorical variables were presented by the frequency of distribution and 95% confidence interval (95%CI). The association between two categorical variables was assessed by the Pearson or Fisher’s chi-square test, when appropriate. The Mann-Whitney test was used for differences of means. The relative risk and the 95%CI were calculated. For all of the analyses, *p*<0,05 was considered significant.

## RESULTS

**Figure 1:**
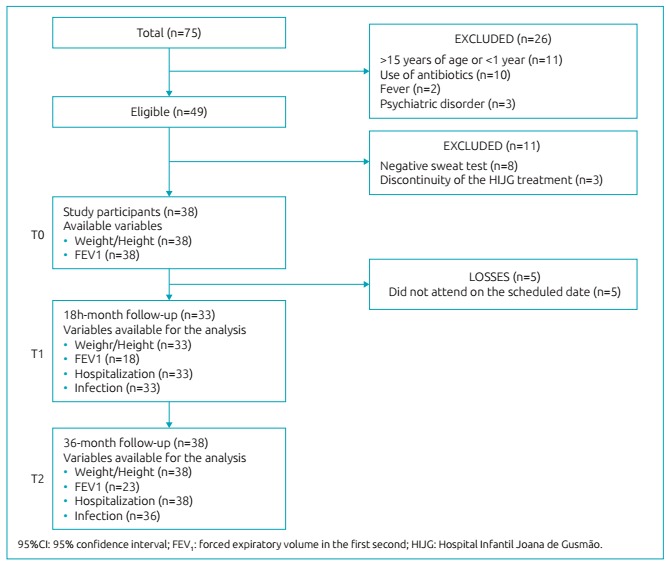
Flowchart of the selection of the study population, constituted of children and adolescents with cystic fibrosis in the clinical follow-up at Hospital Infantil Joana de Gusmão, Florianópolis, Santa Catarina.

At the beginning of the study, of the total of 75 children and adolescents followed-up in the outpatient clinic, 49 were recruited. Of these, 8 had the CF diagnosis ruled out, 2 were transferred to other treatment centers and 1 changed cities. The study remained with 38 children and adolescents (Figure 1).

The median age of the population at the beginning of the study was 3.8 (2.7; 7.0) years. Five were aged <2 years and 17 (44.7%) were male. The homozygous for Delta F508 mutation was found in 18.4% (n=7) of the patients (Table 1).

**Table 1:** Characterization of the children and adolescents with cystic fibrosis in the beginning of the study, Florianópolis, Santa Catarina, 2009 (n=38).

		95%CI
Sex^a^		
Male	17 (44.7)	28.2; 61.3
Female	21 (55.3)	38.7; 71.8
Age (years) ^b^	3.7 (2.7; 7.0)	-
Mutation^a^		
Homozygous Delta F508	7 (18.4)	5.5; 31.3
Heterozygous Delta F508	17 (44.7)	28.2; 61.3
Another mutation	6 (15.8)	3.6; 27.9
Not assessed	8 (21.0)	7.5; 34.6
Culture of oropharyngeal secretion^a^		
Negative	16 (42.1)	25.7; 58.5
*Pseudomonas aeruginosa*	12 (31.6)	11.6; 41.0
Others*	10 (26.3)	16.1; 47.1
Nutritional status^a^**		
Acceptable (>25th percentile)	22 (57.9)	41.4; 74.3
At risk (10-25th percentile)	11 (28.9)	13.8; 44.0
Nutritional failure (<10th percentile)	5 (13.2)	1.9; 24.4
Nutritional status by percentile 50°^a^ **		
<50th percentile	25 (65.8)	45.0; 81.6
≥50th percentile	13 (34.2)	18.4; 50.0
Height/Age^a^		
Low height (<5th percentile)	10 (26.3)	11.6; 41.0
Adequate height (≥5th percentile)	28 (73.7)	59.1; 88.3
Pancreatic insufficiency^a^	29 (76.3)	62.1; 90.5
Shwachman Kulczycki Score^c^	86.8±17.9	80.8; 92.7
%FEV_1_^c^	76.7±19.9	65.6; 87.7
Hospitalization ^a^	14 (36.8)	20.8; 52.9

95%CI: 95% confidence interval; FEV_1_: forced expiratory volume in the first second; ^a^n (%); ^b^Median (interquartile range); ^c^Mean (± standard deviation); *Others: *S. aureus*; *Burkholderia cepacia*; **weight-for-height <2 years; body mass index-for-age ≥2 years by the growth curves from the World Health Organization 2006/2007.

At the beginning of the study, 5 (13.1%) children and adolescents were classified as having nutritional failure. By the WHO (2006, 2007), 25 (65.8%) patients presented percentile <50°; and by the indicator H/A, 10 (26.3%) were classified with low height. After 36 months, 9 (23.7%) patients had nutritional failure according to the WHO curves (2006, 2007), and 27 (71.0%) were below the percentile 50°. There was no significant difference between the prevalence of nutritional failure in the beginning and after 36 months.

The children and the adolescents classified as having nutritional failure at the beginning of the study showed RR 5.00 (95%CI 1.49; 16.76; *p*=0.007) of presenting lung function impairment after 36 months. There was no association between being below the percentile 50° and lung function impairment. The nutritional status was not associated with hospitalization or the presence of positive culture for *P. aeruginosa* (Table 2).

**Table 2:** Bivariate association between nutritional status at the beginning of the study and clinical outcomes after 36 months in children and adolescents with cystic fibrosis, Florianópolis. 2009-2012.

	%FEV_**1**_ ≤70 RR (95%CI)	Hospitalization RR (95%CI)	Positive culture P. *aeruginosa* RR (95%CI)	BMI/Age <50th percentile RR (95%CI)
Nutritional status				
Acceptable	1.00	1.00	1.00	1.00
At risk	1.00	1.14 (0.42; 3.12)	0.45 (0.06; 3.32)	1.66 (1.02; 2.71)*
Nutritional Failure	5.00 (1.49; 16.76)*	1.88 (0.64; 5.53)	1.00	1.83 (0.97; 3.47)
Nutritional status by percentile 50°				
≥percentile 50°	1.00	1.00	1.00		1.00
<percentile 50°	4.61 (0.89; 23.81)	1.91(0.69; 5.23)	-		2.29 (1.37; 3.83)*
Height/Age					
≥percentile 5°	1.00	1.00	1.00			1.00
<percentile 5°	-	1.12 (0.44; 2.84)	0.60 (0.08; 4.29)			0.49 (0.28; 0.86)*

n: absolute number; RR: relative risk; 95%CI: 95% confidence interval; BMI: body mass index; FEV_1_: forced expiratory volume in the first second; * *p*<0.05.

It was observed that children and adolescents who were at nutritional risk presented RR 1.66 (95%CI 1.02; 2.71; *p*=0.037) and were in nutritional failure, RR 1.83 (95%CI 0.97; 3.47; *p*=0.057) of presenting percentile <50° after 36 months. Those classified with low height presented RR 0.49 (95%CI 0.28; 0.86; *p*=0.012) and were below the percentile 50° after 36 months (Table 2).

**Figure 2:**
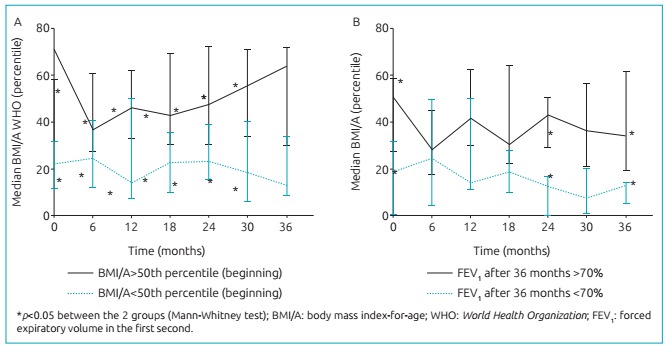
Evolution of nutritional status: (A) stratified in children and adolescents who initiated the study with body mass index-for-age above the percentile 50° (full line) and children and adolescents who initiated the study below the percentile 50° (dotted line), according to the *World Health Organization* 2006/2007 curves; (B) stratified in children and adolescents who presented with forced expiratory volume in the first second >70% at the end of the 36 months of follow-up (full line) and in children and adolescents who presented with forced expiratory volume in the first second <70% at the end of the 36 months.

The children and adolescents who initiated the study above the percentile 50° presented with median of the percentile BMI/A, according to the WHO (2006, 2007), significantly higher than those who initiated it below the percentile 50° at 6 months (*p*=0.028), 12 months (*p*=0.013), 18 months (*p*=0.015), 24 months (*p*=0.036), 30 months (*p*=0.016) and 36 months (p=0.004) (Figure 2 - a1). The children and adolescents who concluded the study without presenting lung function impairment presented higher median of BMI/A, by the WHO (2006, 2007), in the beginning (*p*=0.027), at 24 months (*p*=0.018) and at 36 months (*p*=0.032)(Figure 2b).

## DISCUSSION

In this study, there was an association between the nutritional status failure classified by the WHO curves (2006, 2007), and the lung function impairment. There was no association between the nutritional status and the infection or hospitalization after 36 months.

The nutritional status has an influence on the prognosis of the pulmonary disease of patients with CF. Malnutrition is a result of the increasing energy requirement, the low intake of foods and poor absorption.^17^ The muscle loss and, consequently, the reduction of force and resistance of the respiratory muscles, compromises the diaphragm function, besides compromising the immunity function.^18^ Also, the impaired lung function favors recurring infections, which increases the energy demand, triggering a worse pulmonary situation.^19^

BMI is considered as the most accepted measurement to evaluate the nutritional status in patients with CF. Despite the monitoring and the nutritional orientations, the BMI>percentile 50° can be difficult to reach.^17^ The benefit of the higher BMI in the improvement of the lung function can be attributed to muscle mass.^20^ In a study with 208 children, the lean body mass was associated with better lung function.^21^ The improved nutritional status has a positive influence on pulmonary health and on the global survival. In cases of severe malnutrition in children, there is a major worsening in the lung function, and higher risk of mortality.^6^ Therefore, nutritional evaluations at regular intervals are necessary to identify those at nutritional risk, even before the occurrence of malnutrition.

In this study, the children and adolescents classified with nutritional failure presented risk of developing impaired lung function after 36 months. In a group of 39 individuals with CF, it was observed that the malnourished ones presented with worse pulmonary conditions, according to the parameters of forced vital capacity and FEV_1_.^22^ A similar result was found in a cohort with 3,298 patients aged >2 years, in which, in the cross-sectional analysis, the malnourished patients presented significantly lower mean values of FEV_1_. After 1 year, the malnourished adolescents with reduced W/H had a simultaneous reduction of 16.5% in FEV_1_, whereas those who gained weight in the period had an increase of 2.1% in FEV_1_ (*p*<0.001), emphasizing the relationship between nutrition, lung function and clinical course of CF.^23^

Yen *et al.* ^24^ conducted an observational prospective study using data from the CF Foundation Registry, with 3,142 patients. The authors observed that patients who, at the age of 4, had the indicator W/A>percentile 10° presented better lung function, higher stature and higher survival at the age of 18.

In a cross-sectional study conducted with patients with CF aged between 6 and 18 years, assisted at a reference center in the South of Brazil, the FEV_1_ was significantly associated with BMI (r=0.3; *p*=0.004). BMI/A<percentile 10° was a predictor of low FEV_1_ values. The analysis of regression showed that BMI/A<percentile 10° was associated with the reduction of 25.6% in VEF_1_,^25^ similar to this study, in which there was an association between FEV_1_ and BMI/A.

 In a 4-year longitudinal study with patients in the national Cystic Fibrosis Foundation, in the United States, 968 children aged between 5 and 8 years were assessed. They had pancreatic insufficiency and FEV_1_ between 60 and 140%, observing that the z-score of W/A and the percentage of H/A were significantly associated with changes in FEV_1_ throughout time.^26^

Konstan *et al.* ^4^ conducted a prospective study with patients in the United States and Canada, showing that W/A and H/A were poorly associated with the pulmonary condition at the age of 3, but were strongly associated with lung function at the age of 6. FEV_1_ was higher among those patients for whom W/A had >percentile 10° from the ages of 3 to 6 (FEV_1_ 100±19%), and lower for those who if percentile was <10° (FEV_1_ 84±21%). Data collected prospectively with 319 children, aged between 6 and 8 years, followed-up at a CF center in the United States, showed that, during the follow-up period, the weight gain of 1 kg was associated with the increasing FEV_1_ in 32 mL. The authors concluded that children with higher weight who presented adequate weight gain showed better evolution of FEV_1_,^27^ which was also observed in this study. 

*P. aeruginosa* is known as one of the most important lung pathogens, and the predominant cause of morbidity and mortality in CF. The pulmonary infection in CF is multifactorial, without an isolated cause.^28^ Even though the colonization by *P. aeruginosa* is related with the deterioration of lung function,^25^ the relationship between the infection and the nutritional status is not quite established yet. In this study, there was no association between the colonization by *P. ­aeruginosa* and nutritional status. Similar results were found by Hubert *et al.*,^29^ in a study with 293 adults and 126 children aged more than 7 years, in which there were no differences in the nutritional status of the ones contaminated or not by *P*. ­*aeruginosa*. However, at the end of the follow-up period, only 16.6% (n=6) were infected, therefore the low number of patients infected and the aggressive bacterial eradication are possible reasons why there was no association between the infection and the nutritional status.^30^ Risk factors for the infection by *P. aeruginosa* include sex, and women are more prone to it. The Delta F508 homozygous genotype and the coinfection with other pathogens, such as *S. aureus* and *B. cepacia*, are also independent risk factors.^28^

About 30% of the children and adolescents with CF present low BMI and fat-free mass values. These patients are characterized not only by the reduced lung function, but by the increasing frequency of pulmonary exacerbations and hospitalizations. The literature points out to the association between muscle mass depletion and the increasing number of exacerbations. However, this study did not find a relationship between hospitalizations by pulmonary exacerbation and nutritional status.^31^ However, there are other risk factors that can predispose to pulmonary exacerbations, such as being a woman, having diabetes and worse basal lung function.^32^

It was observed that the children and adolescents classified below the percentile 50° presented risk of having the same classification for the nutritional status after 36 months. The inadequate weight gain and malnutrition are changes commonly found in children and adolescents with CF. In those who present with adequate nutritional status, normal weight gain and growth are expected. Therefore, the early detection of growth below ideal rates allows the early intervention, leading to the indication of the control of weight and height data in a routine basis, at least every three months.^6^ ^,^ ^19^

Regarding the study limitations, it was possible to observe the sample size and the collection of some of the data in the records. However, the recruitment occurred at a reference center for the treatment of children and adolescents aged between 1 and 15 years with CF, in which data are registered based on a protocol previously established by the service, in order to guarantee the quality of the record. Also, for the evaluation of nutritional status, only weight and height were measured, which can limit the adequate evaluation of body composition. Even though there are longitudinal studies that evaluate the impact of nutritional status on the evolution of lung function, in Brazil there are few cohort studies with children and adolescents with CF. Strategies to maintain or recover the nutritional status of children and adolescents with CF should be encouraged and reinforced, as well as the early detection of compromised nutritional status.

In conclusion, the compromised nutritional status was a risk factor for the lung function impairment after 36 months. The nutritional status was not a risk factor for hospitalization and infection by *P. aeruginosa*.
